# Kinematic Analysis-Guided Individualized Exercise for Temporomandibular Disorders: A Case Series

**DOI:** 10.3390/jcm15020655

**Published:** 2026-01-14

**Authors:** Jonggeun Woo, Jeongwoo Jeon, Jiheon Hong

**Affiliations:** 1 Department of Physical Therapy, College of Health Sciences, Sunmoon University, Asan-si 31460, Chungcheongnam-do, Republic of Korea; jgw9968@naver.com (J.W.); srain@sunmoon.ac.kr (J.J.); 2 Digital Healthcare Institute, Sunmoon University, Asan-si 31460, Chungcheongnam-do, Republic of Korea

**Keywords:** temporomandibular disorder, exercise therapy, kinematic analysis, mandibular motion, artificial intelligence

## Abstract

**Background/Objectives**: Exercise-based interventions are strongly recommended for managing temporomandibular disorders (TMDs). However, conventional approaches have limited capacity to address symptoms associated with mandibular kinematic abnormalities and often lack sufficient logical clarity for reproducible clinical applications. Furthermore, although current diagnostic criteria and imaging modalities primarily assess static anatomical conditions, traditional three-dimensional motion analysis is difficult to implement in routine practice. This study aimed to evaluate the effectiveness of a personalized, exercise-based intervention optimized to patients’ lateral excursion (LE) characteristics using an artificial intelligence (AI)-assisted motion analysis system. **Methods**: An AI-based two-dimensional motion analysis platform was used to quantify maximum mouth opening (MMO) and LE in three patients with TMD. Individualized interventions—including massage, stretching, resistance exercises, coordination training, and breathing exercises—were provided over 3 weeks based on each patient’s clinical presentation and movement patterns identified through the kinematic analysis. **Results**: All three patients successfully completed the intervention. Average pain intensity declined across all cases. Mandibular function improved: the mean MMO increased by 38.92% on average, and LE decreased by −1.55 mm on average. **Conclusions**: This study demonstrates that a personalized, exercise-based intervention guided by AI-assisted mandibular kinematic analysis was associated with reductions in pain and improvements in dynamic mandibular function. This approach provides a logically clear and objective framework that may support physical therapy in TMD management, advancing beyond conventional static assessment methods.

## 1. Introduction

Temporomandibular disorders (TMD) are a major cause of non-dental orofacial and head pain, affecting up to 31% of adults, with disk displacement with reduction being the most common subtype [1]. A range of therapeutic modalities is used to manage TMD, from conservative strategies such as pharmacotherapy, splint therapy, and physical therapy to surgical interventions [2,3,4]. Among these, non-surgical conservative approaches, including exercise-based interventions, are strongly recommended because they promote long-term functional recovery and reduce the likelihood of recurrence [5]. However, despite their wide clinical adoption, previous studies indicate that such interventions often lack the logical clarity needed to articulate their conceptual foundations and application principles, limiting reproducibility [6]. The clinical presentation of TMD encompasses diverse symptoms arising from biomechanical disturbances of mandibular motion and pathological changes in surrounding tissues [7]. Common movement abnormalities include deviation, deflection, restricted mouth opening, asymmetric condylar translation, and various forms of head and neck dyskinesia, along with clicking, crepitus, and muscle hypertonicity. To design effective interventions, it is necessary to move beyond a simple listing of these heterogeneous symptoms and instead develop an integrative understanding of the patient-specific pathophysiological and contextual factors that contribute to their manifestation [7,8].

Diagnosis of TMD relies on clinical examination, patient history, and imaging when indicated [9]. However, the widely used Diagnostic Criteria for Temporomandibular Disorders (DC/TMD) remains limited in its ability to capture symptoms beyond pain, joint sounds, and reductions in maximum mouth opening (MMO) [10]. Conventional imaging modalities used in clinical practice including X-ray, computed tomography, magnetic resonance imaging, and electromyography, primarily assess static anatomical structures. Yet, because the temporomandibular joint (TMJ) functions as a ginglymoarthrodial joint, dynamic assessment is essential for detecting alterations in joint kinematics [11]. Three-dimensional motion analysis offers high measurement reliability but is often impractical in routine clinical environments due to its cost, space requirements, and lengthy processing. To address these constraints, artificial intelligence (AI) based markerless motion-tracking technologies have emerged, allowing TMJ movement evaluation using only webcams or smartphone cameras. These systems have demonstrated excellent reliability, with intraclass correlation coefficients exceeding 0.9 compared with conventional three-dimensional analysis, underscoring their strong potential for clinical application [12].

Recent advances in AI-based mandibular motion analysis now make it possible to develop individualized therapeutic exercise programs tailored to each patient’s functional and kinematic characteristics. Algorithm-assisted intervention design directly addresses limitations noted in previous studies, particularly the insufficient logical justification and poor reproducibility underlying many existing therapeutic frameworks [4]. By integrating patient-specific pathophysiological and contextual factors, these systems provide a coherent and structured foundation for planning targeted interventions. Therefore, the aim of this study was to develop an individualized, exercise-based intervention program optimized for the dynamic movement characteristics of patients with TMD using AI-assisted motion analysis and to evaluate both the reproducibility of the intervention and its clinical effectiveness.

## 2. Materials and Methods

This study used a single-subject research design. Participants were recruited between October 2024 and June 2025 through advertisements posted at a local university and on community bulletin boards. Three patients who met the DC/TMD diagnostic criteria were included. Participants were selected based on the presence of persistent TMD-related symptoms, including pain, movement-related deviation or functional limitation in addition to fulfilling the DC/TMD diagnostic criteria. Only individuals aged 20–30 years with the ability to perform the prescribed exercise protocol and attend all assessments were considered eligible. None of the participants had received physical therapy, exercise-based intervention, or invasive treatment for TMD within the 3 months preceding enrollment. Exclusion criteria were as follows: (1) a history of mandibular or craniofacial fracture; (2) current use of non-steroidal anti-inflammatory drugs or other anti-inflammatory analgesics for unrelated medical conditions; and (3) serious systemic comorbidities, including gastrointestinal bleeding, cardiac disease, hepatic disease, or renal disease. The study protocol conformed to the Declaration of Helsinki. All participants received a full explanation of the study procedures, and written informed consent for participation and publication was obtained directly from all participants. Ethical approval was obtained from the Ethics Committee of Sunmoon University (SM–202403–008–1).

All participants underwent two assessments: one at baseline and one immediately after completing the intervention. Assessments included the DC/TMD questionnaire, mandibular motion analysis, and a pain evaluation. Mandibular motion analysis was performed using Google’s Machine Learning Kit for real-time facial feature detection. Accurate landmark tracking and expression analysis were achieved using face-api.js, a JavaScript library built on TensorFlow.js. Smartphone recordings were captured at 60 frames per second using the front camera of a Samsung Galaxy S23 (2023), positioned 50 cm vertically from the participant’s face. From the detected facial mesh, anatomical landmarks corresponding to the nasion, nose tip and chin tip were extracted for kinematic analysis. To convert pixel-based distances obtained from the two-dimensional (2D) recordings into metric units, a subject-specific anthropometric scaling procedure was applied. The physical length of each participant’s nose (measured from the nasion the nose tip using a flexible tape measure) was used as a reference length. The corresponding pixel distance between the same landmarks detected by the facial landmark model was calculated and an individual conversion factor (cm/pixel) was calculated and applied to all mandibular motion measurements. This procedure enabled consistent scaling across recordings. To minimize the influence of head motion artifacts, mandibular movement was analyzed as relative displacement rather than absolute screen coordinates. Specifically, the nose tip was used as a craniofacial reference landmark and mandibular motion was expressed as the displacement of the chin tip relative to this reference. This approach reduced the confounding effects of head translation and pitch during mouth opening. MMO was calculated as the vertical distance between nose tip and chin tip measured the fully closed and fully open mouth positions. Lateral deviation (LD) during mouth opening was defined as the maximum horizontal displacement of the chin tip from the facial midline (nose tip) observed during the opening task. Although this measure does not represent a true lateral border movement, it reflects clinically relevant mandibular deviation and asymmetry patterns commonly observed in patients with TMD. Based on the direction and timing of LD relative to the midpoint of mouth opening (50% of MMO), mandibular movement patterns were classified into 5 kinematic subtypes: straight movement without deviation, initial ipsilateral deviation (IID), late ipsilateral deviation (LID), initial contralateral deviation (ICD) and late contralateral deviation (LCD). Ipsilateral deviation was defined as deviation toward the symptomatic side and contralateral deviation as deviation toward the distinguished side, while initial and late patterns were distinguished by whether deviation occurred before or after 50% of MMO. This classification provided a structured framework for characterizing movement asymmetry and motor control impairment during jaw opening. Within-session repeatability of the proposed 2D analysis pipeline was evaluated by repeatedly measuring mandibular motion variables under identical recording conditions. Measurement consistency and associated error indices were examined to characterize system repeatability.

Pain was assessed using the 10-point numeric rating scale (NRS, 0–10) through participant self-report. The NRS evaluated four pain domains: joint pain (JP), daily-life pain (DP), pressure pain (PP), and referred pain (RP), with intensity based on the average pain experienced during the preceding week. The PP was assessed using a manual algometer (Wagner Force Dial TM FDK 15) applied perpendicularly to the midpoint of the muscle belly in the TMJ region and surrounding muscles, including the masseter, temporalis, and sternocleidomastoid (SCM). Pressure was gradually increased at a consistent rate until the participant verbally indicated the transition from discomfort to pain (e.g., by saying “ah”) or provided a predefined nonverbal signal (hand raising). The pressure value at this point was recorded as the PP. To minimize sensitization effects associated with repeated measurements, PP threshold was measured once per site, and the maximum value was used for analysis.

Cervical function and TMJ sounds were additionally evaluated as part of the clinical assessment to support intervention planning. Cervical posture was assessed using the craniovertebral angle (CVA) to identify forward head posture (FHP) and active cervical range of motion was examined to determine the presence of directional movement restrictions. These findings were used to inform the inclusion and emphasis of cervical posture correction and associated structure management within the intervention. TMJ sounds were assessed during mandibular opening and closing tasks and classified according to their timing of occurrence. Clicking sounds during mouth opening (CMO) were classified as initial CMO when the sound occurred before 50% of maximum mouth opening and as late CMO when it occurred after 50% of maximum mouth opening. Clicking sounds during mouth closing (CMC) were classified when the sound occurred during the mandibular closing phase.

The intervention program aimed to restore TMJ function and reduce pain through an individualized, multidimensional therapeutic exercise protocol delivered over 3 weeks. The program consisted of one weekly 30 min in-person session with a licensed physical therapist, one weekly 30 min remote session for feedback and education, and five home-based exercise sessions per week (minimum of three completed). The intervention framework integrated each patient’s clinical characteristics, such as cervical posture and pain patterns with findings from TMJ motion analysis and comprised 3 primary components delivered across the intervention period.

### 2.1. Intervention Protocol and Procedure

**Figure 1:**
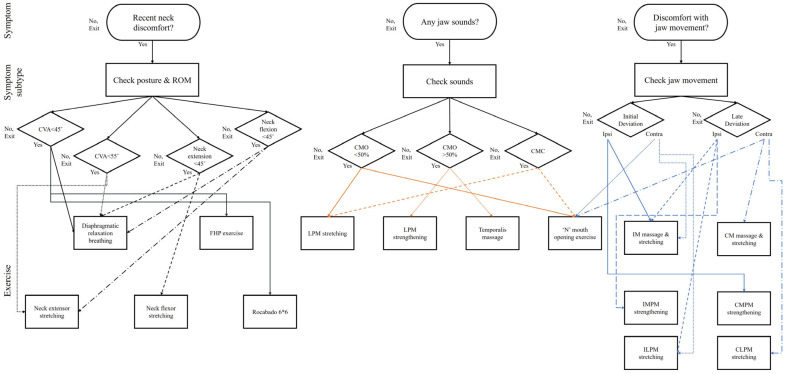
Algorithmic framework integrating mandibular kinematic analysis and clinical features for personalized temporomandibular joint disorder intervention. Figure Legend: The algorithm depicts a structured clinical workflow integrating cervical posture assessment (craniovertebral angle, CVA; range of motion, ROM), joint sound classification during mouth opening (CMO) and closing (CMC), and mandibular deviation patterns (IID, LID, ICD, LCD) to guide individualized exercise selection.

Following the baseline assessment, intervention components were applied according to a predefined procedural framework. Components were delivered in a stepwise sequence, beginning with education and symptom-modulating strategies, followed by associated structure management and manual therapy and progressing to jaw motor control and functional strengthening exercises. Importantly, the intervention algorithm was rule-based and clinician-interpreted, rather than predictive or autonomous. The AI-assisted system was solely to extract and visualize mandibular kinematic features, while all exercise selection, progression, and modification decisions were made by a licensed physical therapist based on predefined clinical rules and patient response. The selection and sequencing of components were determined by each participant’s dominant clinical presentation and mandibular movement characteristics identified through TMJ motion analysis. Across sessions, intervention emphasis and exercise selection were systematically modified in response to changes in cervical posture, joint sounds, pain response, movement quality, and functional performance. This approach allowed progressive adjustment of therapeutic focus while maintaining consistency in overall treatment structure. Figure 1 depicts the conceptual framework of the intervention algorithm, illustrating how mandibular kinematic findings and clinical features were combined to inform individualized treatment strategies.

### 2.2. Patient Education and Lifestyle Management

Patient education focused on avoiding non-functional oral habits and establishing proper postural habits [13]. Educational components included (1) postural hygiene (maintaining appropriate head posture and avoiding sleeping on the symptomatic side), (2) dietary modifications (avoiding hard or chewy foods), (3) oral-habit correction (avoiding gum chewing and excessive MMO and adopting palate-supported yawning), and (4) modification of chewing habits (encouraging variation in chewing direction to reduce habitual patterns).

### 2.3. Associated Structure Management and Manual Therapy

To support functional restoration, cervical posture training and breathing exercises were incorporated. Diaphragmatic breathing was performed in the supine position with minimal upper-thoracic movement, emphasizing bucket-handle motion of the lower ribs at a frequency of 6–10 breaths per minute for 5 min. Cervical posture training included stretching of the SCM and chin-tuck exercises with scapular retraction for patients with forward head posture. When cervical range of motion (ROM) restrictions were present, active ROM stretching toward the restricted direction was applied. Soft tissue manual therapy consisted of ischemic compression to the muscle belly and myotendinous junctions of the masseter and temporalis, performed in three cycles of 10 s of compression followed by 10 s of release. For patients presenting with mandibular movement disorders, selective stretching of the LPM was provided according to the direction of deviation [14].

### 2.4. Jaw Motor Control and Functional Strengthening

Individualized exercise therapy was administered to improve movement patterns and functional strength of the TMJ. Strengthening exercises targeted the medial pterygoid muscle based on the direction of unilateral deviation. Coordination training included controlled opening exercises performed with the tongue positioned posterior to the maxillary incisors, as well as lateral mandibular translation against resistance using a tongue depressor to enhance excursion distance and movement smoothness.

### 2.5. Intervention Adherence and Monitoring

Participant adherence to the intervention was actively monitored throughout the 3-week program. Completion of home-based exercise sessions was self-recorded using a structured exercise log, which documented session frequency and perceived difficulty. These logs were reviewed during the weekly remote sessions to verify adherence and address potential barriers to participation. During the weekly remote sessions participants were asked to demonstrate key exercises in real time via video conferencing. This allowed the physical therapist to directly observe movement quality, confirm correct execution and provide immediate verbal and visual feedback. Exercise intensity and volume were individually adjusted based on participant reported pain levels, perceived exertion and observed movement control to prevent symptom exacerbation. To minimize variability in exercise dosage and ensure intervention fidelity, standardized exercise instructions and visual reference materials were provided. Adherence was defined a priori as completion of at least 3 of the 5 prescribed home-based exercise sessions per week.

### 2.6. AI Statement

During the preparation of this manuscript, the author used AI-based tools solely to assist with automated facial landmark detection and kinematic measurement. All analytical decisions and interpretations were entirely conducted and verified by the author. 

## 3. Results and Discussion

**Figure 2:**
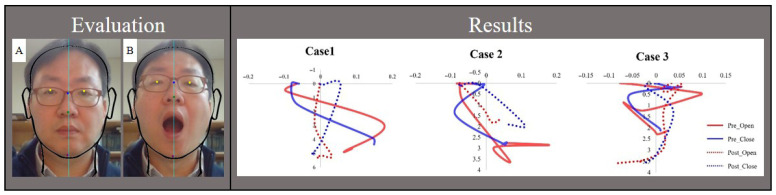
Motion Analysis and case-specific mandibular trajectories during opening–closing movements. Closed mouth (**A**) and Open mouth (**B**).

This study implemented a 3-week individualized exercise therapy intervention based on AI-assisted dynamic movement characteristics in three patients with TMD and evaluated its therapeutic effects. All participants completed core components, including behavioral education, respiratory training, and coordination exercises, supplemented with individualized exercises tailored each patient’s clinical presentation and motion analysis findings. Changes in TMJ functional indicators MMO and LD as well as pain intensity measured by the NRS before and after the intervention, are summarized in Table 1 and Figure 2.

### 3.1. Case 1: Patient with Migraine and Functional Instability

The 21-year-old male patient in Case 1 (height: 185 cm, body mass: 95 kg) presented with migraine and discomfort in the left TMJ, accompanied by a sense of instability during mouth opening. Assessment revealed TMJ clicking, an MMO of 39.8 mm, and a rightward deviation that became pronounced beyond 50% of the mouth-opening range, with an LD of 2.41 mm. Pain levels were reported as JP 5, DP 2, PP 3, and RP 1 on the NRS. The intervention included contralateral masticatory muscle massage and stretching, contralateral LPM stretching, and the “N-opening” exercise. After the 3-week program, the patient reported a subjective reduction in TMJ noise. MMO increased from 39.8 mm to 64.03 mm (gain of 24.33 mm), and LD decreased from 2.41 mm to 0.44 mm (reduction of 1.97 mm). Pain intensity declined across all domains (JP 3, DP 1, PP 2, RP 1). These improvements may be attributed to the effects of masticatory muscle massage, which enhances blood flow in the masseter muscle prone to circulatory insufficiency thereby reducing muscle tension [15]. Stretching of the contralateral LPM likely inhibited excessive muscle activity, suppressed unnecessary movement, and helped restore coordinated activation between the left and right muscles. The combination of resistance exercise and stretching may have increased perfusion in hyperactive and circulation-compromised jaw muscles, contributing to pain reduction and improved mobility. Additionally, stretching facilitates restoration of normal muscle length, reduces muscle tension, and reinforces bilateral symmetry during coordination exercises, which together may explain the observed increase in MMO and reduction in LD [16,17].

In interpreting the marked increase in MMO observed in case 1, individual anthropometric characteristics should be considered. Although previous studies have suggested general reference ranges for MMO in males, more recent evidence indicates that MMO varies with body size, showing positive associations with height and body weight [18,19]. Case 1 presented with a robust physique (height: 185 cm, body mass: 95 kg), which may partly explain the large post intervention MMO observed. Normative data from healthy young adults (American) have reported mean MMO values of approximately 52 mm, with upper values extending beyond 60 mm in individuals with larger body size [20]. Accordingly, while the observed MMO of 64.03 mm should be interpreted with caution, it may still fall within a physiologically plausible range for this patient. These findings do not establish therapeutic effectiveness but suggest that, in individuals with favorable anthropometric profiles, symptom reduction and targeted intervention may be accompanied by substantial functional gains.

### 3.2. Case 2: Patient with Cervical Postural Dysfunction and Pain

The 27-year-old female patient in Case 2 (height: 165 cm, 100 kg) presented with FHP and right-sided TMJ pain, with NRS scores of JP 1, DP 1, PP 4, and RP 2. Functional assessment revealed an MMO of 31.7 mm and an LD of 2.66 mm, demonstrating a late-stage deviation toward the contralateral (left) side. The intervention protocol mirrored that of Case 1 including masticatory muscle massage, stretching, contralateral LPM stretching, and the “N-opening” exercise augmented by cervical stretching to address FHP. Following the 3-week intervention, palpation-related pain decreased by approximately 50%, with NRS scores improving to JP 2, DP 1, PP 2, and RP 2. LD decreased from 2.66 mm to 1.04 mm (reduction of 1.62 mm), reflecting improved movement symmetry. In contrast, MMO decreased slightly from 31.7 mm to 27.64 mm.

In case 2, MMO did not improve and instead showed a reduction, which may be related to the patient’s postural characteristics. Unlike cases 1 and 3, case 2 presented with FHP and cervical-focused intervention was included in the present protocol. However, correction of FHP generally requires integrated interventions targeting the cervical, thoracic and shoulder complex, and cervical intervention alone may be insufficient [21]. Patients with TMD often rely on jaw–head coordination to secure mandibular ROM during mouth opening, and dysfunction in the cervical or upper thoracic regions may constrain MMO [21,22,23]. In this context, the reduction in MMO observed in case 2 may reflect a de-crease in compensatory activation of the SCM muscle following cervical intervention. As cervical muscle tone and postural control were partially normalized, the previously adopted jaw-head compensatory strategy may have been attenuated, resulting in a reduced MMO. Despite this change in MMO, a clear reduction in LD was observed, indicating an improvement in mandibular movement symmetry. Future protocols should incorporate more comprehensive assessment and intervention strategies to address cervical and thoracic postural dysfunction in TMD patients, in addition to targeting mandibular movement symmetry, to facilitate improvements in MMO.

### 3.3. Case 3: Patient with Limited Mouth Opening and Ipsilateral Deviation

The 21-year-old female patient (height: 166 cm, body mass 62 kg) presented with joint sounds in the right TMJ and ipsilateral (left-sided) late-phase deviation. She reported pain levels of JP 2, DP 2, PP 3, and RP 1. Functional assessment revealed restricted mandibular motion, with an MMO of 22.14 mm and an LD of 1.86 mm. Given the ipsilateral deviation, the intervention included same-side masticatory muscle massage and stretching, diaphragmatic breathing exercises, the “N-opening” exercise, ipsilateral LPM stretching, and LPM coordination training. Following the 3-week program, pain levels improved to JP 2, DP 1, PP 1, and RP 1. MMO increased substantially from 22.14 mm to 37.35 mm (gain of 15.21 mm), and LD decreased from 1.86 mm to 0.81 mm (reduction of 1.05 mm), indicating notable improvements in symmetry and functional mobility. These outcomes are likely attributable to reduced hypertonicity and restoration of physiological function of the LPM through stretching and coordination training [16]. The LPM, comprising superior and inferior heads, contributes to mouth opening as well as lateral and protrusive mandibular movements [24,25]. The superior head, which attaches directly to the TMJ disk, plays a key role in disk stabilization. Coordination training of the LPM likely facilitated functional integration of both heads, which may have improved disk control and alleviated movement limitations [24,25].

Existing TMD research has traditionally emphasized increases in MMO as the primary therapeutic objective. More recent literature has emphasized movement quality and symmetry as clinically relevant aspects of TMJ function, with LD representing one observable indicator within this domain [2,6]. The present case series was designed as an exploratory investigation to examine whether selected mandibular motion features could be quantified using an AI-based facial landmark tracking approach and descriptively linked to individualized exercise selection. Within this framework, patient-specific interventions tailored to deviation patterns were associated with observable changes in MMO and LD, suggesting the potential for improvement in mandibular mobility and movement symmetry, along with reductions in pain.

Although the inclusion of only three patients precludes generalization and does not allow conclusions regarding therapeutic effectiveness, this exploratory case series illustrates the potential of integrating AI-assisted motion analysis into the assessment and exercise-based management of TMD. By demonstrating how quantitatively measured mandibular movement features can be incorporated into clinician-guided individualized decision-making, the present findings suggest a possible shift toward data-informed, movement-targeted rehabilitation strategies. As such, this work does not establish a new treatment paradigm but rather opens a conceptual and technical pathway for future research to develop and validate AI-assisted approaches in TMD care.

Across the three cases, changes were observed in MMO, LD, and pain scores following the intervention period. Although causal interpretation is not possible, these descriptive findings suggest that mandibular motion features measured using video-based analysis may support the clinician-guided identification of movement-specific targets and the design of individualized exercise strategies in patients with TMD. In addition, the use of AI-based assessment pipeline indicates the potential feasibility of combining in-person and remote components within a hybrid intervention model. Such an approach may facilitate ongoing monitoring of movement characteristics and patient engagement beyond the clinical setting, while preserving clinician-led decision-making in personalized rehabilitation.

Despite these exploratory observations, several methodological and technical limitations require careful consideration. The absence of a control condition, repeated baseline measurements, and follow-up assessments precludes causal inference, and the small, clinically selected sample limits generalizability. From a technical perspective, reliance on 2D kinematic analysis constrains characterization of out of plane mandibular motion and may be sensitive to head movement, camera positioning, and environmental conditions. In the present study, head motion was not physically restricted during data acquisition. Although analytical head motion correction was applied within the 2D AI-based framework, residual error is likely to remain, particularly given the inherent limitations of two-dimensional kinematic analysis. Furthermore, the current study does not include formal validation of the measurement pipeline against an external reference standard or reliability testing under varied clinical scenarios. The AI-based component was confined to automated facial landmark detection and kinematic feature extraction, with all intervention decisions guided by clinical judgment rather than predictive modeling. Future studies should employ controlled study designs with larger samples, predefined decision rules, validated measurement systems, and more robust strategies for head motion stabilization or compensation to establish reproducibility, measurement validity, and clinical utility.

In conclusion, this study demonstrates the feasibility and clinical value of quantifying dynamic mandibular motion, including LD, and using these metrics as therapeutic targets. This approach holds promise for development into an AI-integrated predictive framework for individualized exercise prescription and functional assessment.

## Figures and Tables

**Table 1:** Case-specific changes in mandibular function and pain variables.

Case	Age/Sex	Symptoms	Variable	Baseline	Post-Intervention (Week 3)	Difference
Case 1	21/M	Migraine, left TMJ pain, clicking sound, anxiety, LCD	MMO (mm)	39.80	64.03	24.23
			LD (mm)	2.41	0.44	−1.97
			JP/DP/PP/RP (NRS)	5/2/3/1	3/1/2/1	−2/−1−/1/0
Case 2	27/F	FHP, right TMJ pain, LCD	MMO (mm)	31.70	27.64	−4.06
			LD (mm)	2.66	1.04	−1.62
			JP/DP/PP/RP (NRS)	1/1/4/2	2/1/2/2	−1/0/−2/0
Case 3	21/F	Right TMJ clicking sound, left late deviation, MMO limitation, IID	MMO (mm)	22.14	37.35	15.21
			LD (mm)	1.86	0.81	−1.05
			JP/DP/PP/RP (NRS)	2/2/3/1	2/1/1/1	0/−1/−2/0

M, male; F, female; TMJ, temporomandibular joint; LCD, Late contralateral deviation; IID, Initial ipsilateral deviation; FHP, forward head posture; MMO, maximal mouth opening; LD, lateral deviation; JP, joint pain; DP, daily pain; PP, pressure pain; RP, referred pain; NRS, numeric pain rating scale. Difference values represent directional changes calculated as post-intervention (week 3) minus baseline values. For pain variables, negative values indicate symptom reduction, whereas positive values indicate increased pain.

## Data Availability

The data presented in this study are available on request from the corresponding author. The data are not publicly available due to privacy restrictions.

## Abbreviations

The following abbreviations are used in this manuscript:

AI	Artificial intelligence
DP	Daily-life pain
FHP	Forward head posture
ICD	Initial contralateral deviation
IID	Initial ipsilateral deviation
JP	Joint pain
LCD	Late contralateral deviation
LD	Lateral deviation
LID	Late ipsilateral deviation
LPM	Lateral pterygoid muscle
MMO	Maximum mouth opening
NRS	Numeric rating scale
PP	Pressure pain
ROM	Range of motion
RP	Referred pain
TMJ	Temporomandibular joint
2D	Two dimensional
